# Supplementary material for: Projecting COVID-19 disease severity in cancer patients using purposefully-designed machine learning
Source: BMC Infect Dis. 2021 May 4;21:391. doi: 10.1186/s12879-021-06038-2 (PMC8092998; doi:10.1186/s12879-021-06038-2)
Supplement: Supplementary file 1 — Additional file 1. [file 12879_2021_6038_MOESM1_ESM.docx]

**Table S1**: List of all 267 variables. Bolded features are the subset of 55 used in our analysis.

1. Basic patient variables (6).

Sex, **Race**, **Ethnicity**, **Age**, **Smoker**, **BMI**

2. Labs (27): Laboratory work variables.

**ALT**, APTT, **AST**, Absolute Lymphocytes, **Absolute Neutrophils**, **Absolute Reticulocytes**, **Albumin**, **BUN**, Total Bilirubin, **C-Reactive Protein**, **Creatinine**, **D-Dimer**, **Erythrocyte Sedimentation Rate**, **Ferritin**, **Glucose**, **HGB**, **Interleukin 1 beta**, **Interleukin 10**, **Interleukin 6**, **Lactate Dehydrogenase**, **PT**, **Platelets**, **Procalcitonin**, **Troponin I**, WBC, E6, **ANC-ALC-ratio**

3. Cancer-related (26): Each variable is binary, indicating whether the patient has the cancer or is taking a cancer-related medication.

**Bladder, Bone Marrow, Breast,** Conn, Endometrium, **GI, GU,** Kidney, **Leukemia, Lung, Lymph Nodes, NHL (Non-Hodgkins Lymphoma), PCD (Plasma Cell Dycrasias),** Pancreas, Prostate, Rectum, Sigmoid Colon, Skin, Thyroid

Active Cancer: Whether the patient received radiation, chemotherapy, or surgical intervention in the last 6 months.

**Check-point inhibitors**: Whether the patient is taking one of: pembrolizumab, nivolumab, atezolizumab, avelumab, durvalumab, ipilimumab.

**Corticosteroids**: Whether the patient is chronically on prednisone, prednisolone, or dexamethasone. Chronic is defined as > 5 mg of prednisone (or equivalent) for 5 days.

Group**: Hematologic, HCT, Solid**

4. ICD codes for pre-existing diagnoses (195): The first two characters of the ICD-10 code. Each variable is binary, indicating whether the ICD code was assigned to the patient. Examples include:

**E1**: diabetes mellitus

**I1**: hypertensive diseases

**I4**: cardiac disorders

**J4**: chronic lower respiratory diseases

**Major Surgery**: Whether the patient had a major surgery in the last 30 days that required general anesthesia.

5. Radiology (13): Patient’s X-rays were classified as “normal”, “abnormal” or “indeterminate”. When available, current studies were compared to prior to ensure findings were new. If patients prior X-rays demonstrated findings, these were classified as having “background disease”. If no findings were detected on the X-ray or no new findings were noted when compared to prior, the study was determined to be “normal”. Studies with extensive background metastasis, large neoplasms, effusions or post treatment changes, which would significantly obscure new findings, were classified as “indeterminate”. A study was determined as “abnormal” if airspace and/or reticulonodular opacities were noted. Airspace opacities were divided into patchy opacities and segmental or lobar consolidations. Findings were further classified as “unilateral” or “bilateral” and as predominantly in the inferior lobes (“gradient”) or diffuse involving upper lobes. In addition, pleural effusions were recorded and classified by amount (small, moderate or large).

**Normal**

**Abnormal**

**Indeterminate**

**Background disease**

**Unilateral**

Bilateral

Airspace Opacities

Patchy Opacities

**Consolidations**

Gradient (1. basilar, 2. upper and/or lower)

**Reticulonodular Opacities**

**Effusions**

Amount (1. small, 2. moderate, 3. large)
